# Supplementary material for: Are carbon emissions trading and green financial instruments synergistic? -Comprehensive quantitative research based on content analysis
Source: PLoS One. 2024 Mar 7;19(3):e0298601. doi: 10.1371/journal.pone.0298601 (PMC10919601; doi:10.1371/journal.pone.0298601)
Supplement: S1 Data — (ZIP) [file pone.0298601.s001.zip › Data/地级市Z_F工作报告.zip!/┴╔─■/╜⌡╓▌╩╨/└┤╫╘╣1⁄2╓┌║┼í╛┬φ┐╦╩2╛▌═°í┐_2011.docx]

各位代表：

　　现在，我代表市政府向大会报告工作，请予审议，并请市政协各位委员提出意见和建议。

　　一、2010年工作总结和“十一五”回顾

　　2010年，在省委、省政府和市委的正确领导下，我们紧紧咬定“两个不低于”的发展目标，全力推进沿海、县域、城区三大经济区域协调发展，辽西沿海经济区中心城市建设实现重大突破，圆满完成了市十四届人大三次会议确定的各项目标和任务。

　　预计全市地区生产总值完成916亿元，增长17%；全社会固定资产投资完成700亿元，增长100%；规模以上工业增加值完成430亿元，增长18%；地方财政一般预算收入完成81亿元，增长38.9%；社会消费品零售总额达到330亿元，增长18.9%；实际利用外资5亿美元，增长36%；实际利用域外资金400亿元，增长86.9%；出口总额11亿美元，增长36.4%；城镇居民人均可支配收入17000元，增长11%；农民人均纯收入7422元，增长12%。

　　（一）沿海经济带建设日新月异

　　沿海经济带“五大板块”基础设施建设和产业发展齐头并进，大开发大开放格局全面拉开。

　　锦州港完成港建投资21亿元，创历史新高。港口吞吐量达到6006万吨、集装箱运输70万标箱。209、210泊位建成投入试运行，中电投专业化煤炭码头、龙栖湾港区开工建设。

　　锦州经济技术开发区升级为国家级开发区。国家石油战略储备基地、中北石化60万吨润滑油等一批重点项目取得实质性进展。白沙湾行政生活区建设快速推进，滨海体育中心、职教园区等一批重点工程进展顺利。

　　龙栖湾新区获批省级开发区。基础设施建设全面推进，城际铁路、疏港铁路纳入省部计划，龙栖湾大道建成通车。年产240万吨差别化纤维、台湾光电产业园、锦州旭龙太阳能产业基地等一批重大项目开工建设，光伏和石化轻纺“双千亿”产业基地全面启动。2013中国锦州世界园林博览会项目获批筹建。

　　大有经济区起步区实现“七通一平”，中冶京诚标准厂房、科诚电阻器、光华航模发动机等项目全面推进。

　　建业经济区规划和航空物流城规划初步完成，锦州湾机场选址获国家批准，航站区回填工程全部完成。

　　（二）工业经济质量效益同步提升

　　全年共实施亿元以上重点工业项目70个，完成固定资产投资280亿元，增长45%；规模以上工业企业达到979户，增加76户。

　　石化、铁合金、农产品深加工等传统支柱产业快速发展，光伏、汽车及零部件、精细化工等新兴支柱产业不断壮大，石化公司130万吨加氢裂化、中信锦铁中低碳锰铁扩能改造、锦州佑华硅2000吨单晶硅、锦恒汽车安全系统产业化、辽宁天合1.9万吨织物整理剂等一批重点项目竣工试运行，全年实现光伏产业产值100亿元、汽车及零部件产值70亿元、精细化工产值85亿元。

　　科技创新体系进一步完善，企业自主创新能力快速提高。全口径完成专利申请539项，65个科技项目列入国家和省科技计划，新增省级工程技术研究中心3家、省级企业技术中心4家、国家高新技术企业11家。规模以上企业实现高新技术产品增加值90亿元，增长26.9%。

　　中小企业融资难题有效缓解，12家担保机构累计担保企业37户，12家小额贷款公司获准开业。

　　节能减排成效明显，5个项目获省节能专项资金支持，9座污水处理厂全部建成运行，万元GDP综合能耗下降5%，全年减排二氧化硫2.6万吨、化学需氧量2.1万吨，城区空气质量优良率达到95.6%。

　　（三）农业和县域经济发展势头迅猛

　　现代农业建设快速推进，五大产业结构和五大运营体系不断完善。粮食总产量达到36.4亿斤。新增设施农业面积20.3万亩、畜禽标准化养殖小区350个、水产健康养殖示范区40个。新增产业化龙头企业78户。订单农业面积达到260万亩，“三品”生产基地认证面积达到252万亩。农业生产综合机械化水平达到65%。农民专业合作社发展到820家。

　　农村生产生活条件进一步改善。新增灌溉面积11.4万亩、水土流失治理面积27.5万亩、改善涝区面积17万亩。新增耕地6万亩，耕地总量继续保持正增长。重大动物疫情连续五年零发生，畜产品安全水平全国领先。气象预报预警、防灾减灾能力明显提高。锦凌水库水源涵养林、可视荒山绿化等8项生态工程快速推进，人工和飞播造林40万亩。新建“一池三改”沼气池1.4万个、农村饮水安全工程144处。新建乡镇客运站7座，县(市)乡公路大中修工程顺利完工。完成整村推进项目32个，3.9万贫困人口温饱问题稳定解决。

　　县域工业园区基础设施建设日趋完善，产业集群发展势头强劲，“一县一业”全面确立。庞河、沟帮子、七里河、大有等园区累计投入基础设施建设资金30亿元，“五通一平”建成区面积达到22.6平方公里，累计入驻企业150户。国大乳业12座现代化奶牛养殖场项目已有6座建成投入使用，30万吨液态奶项目竣工试生产。县域地区生产总值增长22%、全社会固定资产投资增长78%、地方财政一般预算收入增长61%。凌海地方财政一般预算收入在全省排名第十，黑山、北镇、义县分别升至31位、36位、37位，第一轮“双增升位”目标胜利完成。

　　县城改造和扩建工作全面启动，中心乡镇建设加快推进，黑山八道壕煤矿棚户区改造创造了当年搬迁、当年建设、当年交付使用的辽宁棚改新速度。

　　（四）城区经济和服务业蓬勃发展

　　城区经济“三年倍增”计划成效显现，地区生产总值增长20%，地方财政一般预算收入增长34.4%，固定资产投资增长50%，域外资金到位额增长92%。

　　现代服务业发展实现新的突破，中央大街十里商街改造控制性详规已经完成，重点项目建设及老城区开发改造全面启动。锦州国际酒店、国际会展中心项目竣工。地一大道、茂业百货、亿隆国际广场、家乐汇广场等33个商业项目开工建设，太阳广场城市综合体项目开始拆迁，港口局、“八大局”及周边城市综合体项目正在洽谈推进。改造“万村千乡市场工程”农家店1474家，农村消费市场日益活跃。农展会、古玩节、房交会等大型展会收效明显。全年完成房地产开发850万平方米，投资93亿元，增长88%。凌海大有温泉旅游风情小镇和义县七里河温泉旅游度假村项目列入省首批重点开发项目，凌河区紫荆温泉度假村项目列入市区重点开发项目。锦州银行在哈尔滨、丹东设立分行。全市金融机构存贷比达到62%。

　　（五）改革开放实现重大突破

　　抢抓国家战略机遇，坚持以沿海经济带产业园区和县域工业园区建设为平台，积极开展“走出去”和“请进来”招商活动，中国光伏之都、中央大街十里商街、中国海洋城等主题概念招商和锦州湾投资座谈会、锦州光伏产业国际高峰论坛、天津招商引资项目推介会等活动在国内外产生广泛影响，越来越多的央企、知名大企业集团开始看好锦州、投资锦州。

　　充分利用国家和省鼓励出口政策，积极调整出口产品结构，大力支持企业开拓国际市场，确保了外贸出口稳定增长。锦州正式成为国家加工贸易梯度转移重点承接地，再一次获得“国家级名片”。

　　国企改革稳步推进。35户企业完成改制任务，5户企业政策性破产进入法律程序，9户企业遗留问题得到彻底解决。热电总公司调整生产经营结构进展顺利。燃气总公司和污水处理厂股份制改造全面完成，率先在省内实行特许经营制度。

　　（六）民生和各项社会事业成就显著

　　就业再就业态势良好。网上创业工作全面推开，成功组建了网商创业孵化园。完成普惠制培训3.8万人、实名制安置就业5.6万人，零就业家庭始终保持动态为零，登记失业困难家庭大学生当年100%实现就业，城镇登记失业率控制在3.8%以内。

　　社会保障体系日趋完善。基本养老、医疗、失业、工伤、生育参保人数分别达到60.3、118.9、37.7、27、16.5万人。城乡低保和农村五保供养标准提标工作顺利完成。新建区域性中心敬老院5所，新增城乡社区公共用房5000平方米，100个农村社区服务中心建设任务完成过半。保障性住房建设稳步实施，新增实物配租1769户、租赁补贴1348户、经济适用住房2500户。

　　文化教育体育事业全面发展。开发区“双高普九”通过省评估验收。新建农村九年一贯制学校11所，完成危房改造2万平方米。新华书店转企改制加入北方传媒取得阶段性成果。4家博物馆和纪念馆全部实现免费开放。农家书屋三年建设任务全部完成。大型舞蹈诗《大辽西》获省第二届文化艺术政府文华奖，市图书馆获“国家一级图书馆”称号。“辽西木偶戏”、“锦州满族民间刺绣”等国家和省级非物质文化遗产集体亮相上海世博会。广播电视主题报道、舆论监督和大型宣传活动影响广泛。圆满承办了省第七届少数民族传统体育运动会，取得金牌总数、奖牌总数、团体总分第二名的优异成绩。

　　医疗卫生服务水平不断提升。农村三级医疗服务体系进一步健全，新农合筹资标准提高到每人每年150元。医药卫生体制改革稳步推进。医疗信息共享平台建设成效明显，“一个中心、多个专科”建设逐步深化。公共卫生均等化、疾病预防控制、卫生应急体系建设不断加强。深入开展“生育关怀行动”和“救助贫困母亲行动”，计划生育贫困家庭女孩和贫困母亲得到有效救助。人口出生率控制在5.96‰。

　　城市建设管理全面推进。锦凌水库建设初具雏形，一、二期移民工作正式启动。云飞大桥建成通车，广州街公铁立交桥、中环路三期续建等工程顺利推进。市府路、云飞街等11条街路新建大修改造任务如期完成。小扒沟带状公园、云飞南街等新建绿化工程和43条街路、14个公园广场、8个街心绿地补植工作全部完成。接收社会自管二次泵站13座，拆除并网小锅炉45台。“门前三包”和街路网格化管理进一步加强。

　　和谐锦州建设取得新成就。锦州获得“中国十佳和谐可持续发展城市”殊荣。第六次全国人口普查取得阶段性成果。圆满完成“五五”普法任务。人民调解工作取得新成绩。成功办理法律援助案件3477件。强化产销衔接，加强价格监管，物价保持总体平稳。公安信息化建设步伐加快，新装动态视频监控探头2.6万个，社区巡逻与街面武装巡逻、警务工作站实时查控联动防控格局全面形成，社会治安防控体系建设进一步加强，打击刑事犯罪成效显著，群众安全感普遍增强。市、县两级信访大厅建成投入使用，一批信访积案妥善化解，越级上访量“减量退位”。安全生产工作扎实有效，继续保持了安全生产的平稳态势。

　　（七）政府自身建设进一步加强

　　自觉接受人大法律监督、政协民主监督和群众的社会监督，坚持定期向市人大和市政协报告、通报工作，积极采纳各民主党派、工商联、无党派人士意见。全年共办理市人大代表建议和政协提案378件，见面率、满意率均达100%。

　　积极推进法治政府建设，加大政务公开工作力度，公开范围逐步向街道、社区、乡镇延伸。加强政府机关作风建设，狠抓纠风专项治理和行政效能监察，公共管理和服务水平进一步提升。

　　拥军优属、军民共建活动深入开展，国防动员、民兵预备役工作成绩显著。国家安全、民族宗教、妇女儿童、外事侨务、防震减灾工作不断加强。审计、统计、质监、档案、对台、残联、慈善等工作取得新成绩。

　　各位代表，经过全市干部群众的艰苦创业和无私奉献，“十一五”目标任务已经胜利完成。五年来，在省委、省政府和市委的正确领导下，我们紧紧抓住东北老工业基地振兴、辽宁沿海经济带建设等重大历史机遇，克服国内外复杂多变外部环境的不利影响，全面贯彻科学发展观，深入实施“一纲五线”发展战略，聚精会神搞建设，一心一意谋发展，开创了经济平稳较快增长、民生不断改善、社会持续稳定的崭新局面，为锦州实现跨越发展奠定了坚实基础。

　　过去的五年，是综合经济实力大幅跃升、辽西沿海经济区中心城市地位日益凸显的五年。2010年，全市地区生产总值是2005年的2.6倍，地方财政一般预算收入是2005年的3.6倍，总量均位居全省第五位。五年完成的全社会固定资产投资是“十五”时期的4.5倍。锦州正在加速实现由区位型中心城市向经济型中心城市、内陆型城市向现代口岸城市的根本转变。

　　过去的五年，是产业结构不断优化、县域和城区经济迅猛发展的五年。三次产业比重调整为16.4∶49.1∶34.5，第二产业增加值提高16个百分点。规模以上工业增加值年均递增20.2%。高新技术产业增加值年均增长27%。县域经济“农业升级”、“双增升位”任务如期完成。现代服务业快速提档升级，城区发展活力开始迸发。

　　过去的五年，是改革开放不断深入、城乡面貌日新月异的五年。累计完成国有企业改制215户。减少和规范行政审批340项。新批外商投资企业是“十五”时期的2倍。实际利用外资年均增长49.7%。老城区改造和城市南扩工程全面启动。滨海大道等一批重大基础设施建设项目建成投入使用。全市1109个村全部实现村通油路的目标。

　　过去的五年，是以民生为重点的社会建设突飞猛进、人民生活质量显著提高的五年。创业带就业、养老保险扩面等民生工程相继实施，“五大社会保险”框架全面形成，以医疗救助、社会互助和灾民救济等制度为配套的城乡社会救助体系初步建立。城镇居民人均可支配收入和农民人均纯收入分别是2005年的1.9倍和2倍。

　　过去的五年，是精神文明与民主法制建设全面加强、政府系统行政效能显著提升的五年。全面推行依法行政五年规划。强化公共服务管理，不断深化政务公开，完善维护稳定长效机制，深入推进矛盾纠纷化解，群众合法权益得到有效维护。切实加强软环境建设，积极开展“十学大连”活动，全面弘扬“勇于牺牲、敢为人先”的锦州精神，政府系统作风建设明显加强。

　　各位代表，“十一五”时期锦州发生的巨大变化令人鼓舞，积累的宝贵经验弥足珍贵，奠定的坚实基础影响深远。回顾2010年和“十一五”时期的工作，我们深切感受到，锦州取得的一系列重大成就，是省委、省政府和市委正确领导的结果，是全市人民团结一心、共同奋斗的结果。在此，我代表市政府向在各个领域和岗位上辛勤劳作、做出贡献的全市人民表示崇高的敬意！向给予政府工作大力支持的人大代表、政协委员、各民主党派、工商联和各人民团体，向始终关心和支持政府工作的离退休老同志，向中省直各部门、各单位，向驻锦部队和武警官兵，向所有关心支持锦州建设和发展的海内外朋友，表示衷心的感谢！

　　我们在为“十一五”取得的成绩感到欣喜的同时，也清醒地认识到，当前锦州经济社会发展还面临一些困难和问题。主要是经济总量偏小、结构不尽合理，节能减排任务仍很艰巨，环保、土地、融资等发展瓶颈问题还很突出，城乡居民生活水平同发达地区相比还有较大差距，社会仍存在一些不稳定因素，政府自身建设还有待进一步加强。这些困难和问题，我们将在今后工作中认真加以解决。

　　二、“十二五”展望和2011年工作安排

　　“十二五”时期，是锦州的战略机遇期和黄金发展期，做好今后五年工作，对锦州经济社会的大发展、快发展具有决定性意义。

　　“十二五”时期，全市经济社会发展的总体要求是：全面贯彻落实党的十七届五中全会、中央和省经济工作会议、市委十届十次全会精神，以科学发展为主题，以加快转变经济发展方式为主线，深入实施“一纲五线”发展战略，以增量带动结构优化，以创新促进产业升级，以全民创业增强发展活力，以改善民生满足人民群众的新期待，着力打造新欧亚大陆桥“桥头堡”，把锦州建设成环渤海地区特大型港口城市、辽西沿海经济区中心城市和中国光伏产业之都，确保实现进入全省第一方阵的奋斗目标。

　　“十二五”时期，我们要坚持“两步走”发展战略，即：到2012年，全市地区生产总值、规模以上工业增加值、全社会固定资产投资、社会消费品零售总额、地方财政一般预算收入、实际利用外资要在2009年基础上翻一番，全面实现“三年倍增”计划；再经过连续3年的不懈奋斗，到2015年，全市地区生产总值达到2300亿元，年均增长20%，三次产业比发展到10∶50∶40。

　　“十二五”时期，我们要牢牢把握以下四个方面的工作原则：

　　一是坚持科学发展，始终以科学发展观为统领，优化经济结构，转变发展方式，破解发展难题，走生产发展、生活富裕、生态良好的可持续发展道路，实现经济社会又好又快发展。

　　二是坚持率先发展，紧紧抓住辽宁沿海经济带、国家级开发区、全国加工贸易梯度转移重点承接地等重大机遇，推进沿海、县域、城区三大经济区域快速协调发展，全面提升锦州在全省的位次，确保稳定进入全省第一方阵。

　　三是坚持创新发展，实施科技兴市和人才强市战略，加强科技创新体系建设，完善科技创新激励机制，培育壮大创新主体，优化创新发展环境，增强自主创新能力，建设创新型城市，使创新发展成为锦州振兴的首要推动力量。

　　四是坚持和谐发展，更加注重社会建设，着力保障和改善民生，真正把加速发展的出发点和落脚点体现在富民、惠民、利民上，不断满足全市人民日益增长的物质文化生活需要，加快提升全市人民的生活质量和幸福指数。

　　各位代表，今年是实施“十二五”规划的开局之年，也是实现“三年倍增”计划、稳定进入全省第一方阵的关键之年，做好今年的工作，意义重大，责任重大，影响深远。

　　今年全市总体奋斗目标是：主要经济指标要确保进入全省前5名，综合经济实力要确保进入全省第一方阵。

　　围绕这一总的奋斗目标，今年全市地区生产总值要实现1100亿元，增长20%；地方财政一般预算收入实现115亿元，增长40%；全社会固定资产投资实现1200亿元，增长70%；规模以上工业增加值实现560亿元，增长30%；社会消费品零售总额实现400亿元，增长20%；实际利用外资10亿美元，增长100%；实际利用域外资金700亿元，增长75%；出口总额13亿美元，增长18%；城镇居民人均可支配收入和农民人均纯收入分别增长16%和17%。

　　为确保上述目标的完成，我们要突出抓好以下七个方面的重点工作：

　　(一)全方位扩大对外开放

　　不断强化大开发大开放意识。我们要牢牢把握国家加工贸易梯度转移重点承接地和国内外产业转移的重大历史机遇，积极承接东南沿海及日本、韩国、台湾等地的产业转移，引进资金，引进项目，引进先进的团队，引进成功的发展模式，用锦州的开放建设开放的锦州。

　　切实加大招商引资工作力度。这是加大投入的主渠道，是实现锦州跨越发展的必然选择。要坚持招商引资中心地位不动摇，深入开展项目年活动，一切围绕项目转，一切围绕项目干，一切依据项目看，在全市掀起项目洽谈、签约落地、开工建设、达产达效、做大做强五个高潮。紧紧盯住世界500强企业、央企、行业龙头企业和国内外重点地区，突出抓好新兴支柱产业、县域主导产业集群招商和以重点园区为平台的主题概念招商，着力引进一批具有牵动发展全局作用的大项目、产业集群项目、高科技项目、著名企业和驰名品牌项目，形成锦州快速发展的强大后发优势。

　　牢固树立抓项目、抓投入就是推进锦州快速发展的理念。突出抓好省委、省政府重点推进的重大项目，突出抓好事关锦州全局的重大项目。今年全市共安排基础设施、工业、商贸物流、房地产、能源、交通、农业、社会事业和民生工程八个方面的亿元以上项目435个，总投资2200亿元，其中上年结转项目95个，今年新开工建设项目340个，力争完成投资1500亿元。对这些重点项目，要建立领导和部门承包责任制，明确标准，明确责任，明确完成时限，确保项目落实到位、资金投入到位，用实实在在的项目、实实在在的投入推动锦州经济社会的大开发、大开放和大发展。

　　以大开放推进工业五项工程的全面提升。企业提升工程，要突出抓好中信锦铁15万吨高碳铬铁、哈啤搬迁扩产等6个技改扩能项目，新增销售收入50亿元以上企业1户、销售收入10亿元以上企业5户。项目工程，要确保博阳光伏100MW组件、华昌光伏科技200MW单晶硅太阳能电池等24个项目建成投产，锦州石化公司25万吨烷基化装置、天合精细化工60万吨润滑油等32个项目开工建设。产业集群工程，要确保实现汽车及零部件产值100亿元、精细化工产值130亿元、光伏产业产值150亿元、特种铁合金产值400亿元。并购工程，要确保完成并购项目4个，争取4户企业认定为省级技术中心。节能降耗与淘汰落后产能工程，万元GDP综合能耗和主要污染物排放量要降低5%以上，城区空气质量优良率达到96%以上。今年，工业经济占三次产业比重要达到50%以上，成为全市经济的主体和支撑。

　　(二)加速构建沿海经济带大开发新格局

　　充分利用国家战略和国家、省级开发区的政策效应，在“五大板块”建设上实现重大突破。

　　锦州港，要突出抓好15万吨级航道、三港池通用泊位等基础设施和龙栖湾港区防波堤工程建设，开工建设华润电力煤炭码头，确保完成港建投资15.3亿元，港口吞吐量达到7000万吨，集装箱运输80万标箱。

　　经济技术开发区，要突出抓好800万吨重油和200万吨道路沥青、国家石油战略储备基地、蒙古贞热电厂、中北石化60万吨润滑油等一批重点项目，确保年内开工建设。推进锦赤铁路建设工程顺利实施。加大白沙湾行政生活区开发建设力度，开工建设CBD中央商务区和辽宁省农业经济学校新校区，完成滨海大道绿化亮化、滨海体育中心主体和滨海医院一期工程，确保职教园区竣工投入使用。加快推进以钛业为主导的汤河子特种金属产业基地建设进程，力争10个亿元以上项目落户园区。

　　龙栖湾新区，要加快光伏、石化轻纺“双千亿”产业基地和“中国海洋城”建设步伐，确保工业起步区全部实现“七通一平”，9个以上工业项目建成投产，完成房地产开发130万平方米。加快启动实施2013中国锦州世界园林博览会项目，加速打造独具海洋特色的中国北方风景园林。

　　大有经济区，要突出抓好园区配套设施建设，力促中信锦铁20万吨钛白粉、中广核太阳能电池等重大项目落户园区，确保科诚电阻器、光华航模发动机等项目竣工投产，京沈高速凌海出口至滨海大道19.8公里一级路建成通车。

　　建业经济区，要突出抓好经济区总体规划、起步区控制性详规和15平方公里起步区基础设施建设，开工建设锦州湾机场和航空物流城。

　　(三)强力推进县域经济跨越发展

　　加速推进县域工业化、城镇化和农业产业化进程，用“三化”推动“三农”工作，全面建设社会主义新农村，加速城乡一体化进程。

　　在工业化上，进一步加大县域工业园区基础设施建设投入力度，确保每个园区资金投入比去年增长20%以上；做强做大电动车、工业炉、电力电器、塑料化工等县域产业集群。今年，每个县(市)至少要打造一个产值超百亿元的产业集群。

　　在城镇化上，加强道路、给排水、集中供热等基础设施建设；突出抓好房地产开发，县域房地产施工面积要达到350万平方米以上；启动7镇、5村小城镇建设试点工作，增强小城镇对农村人口的吸纳能力。

　　在农业产业化上，突出抓好“一县一业”，新增设施农业面积8万亩；新增种植业“三品”认证40个、面积50万亩；新增规模以上农业产业化龙头企业44家，总量达到380家；农业机械化水平达到67%；畜牧业产值占农业总产值50%以上，肉蛋奶产量稳居全省前列；切实加强疫情监控和应急管理体系建设，坚决防止重大疫情发生。

　　今年，县域地区生产总值增幅要在30%以上，财政一般预算收入增幅要在50%以上。凌海要力争在“十二五”时期进入全国百强，黑山、北镇、义县在全省位次要明显前移。

　　(四)全面提升中心城市的核心竞争力

　　以争创国家园林城市、国家卫生城市和全国环保模范城市为重点，全面提升城市建设管理水平，树立锦州城市的崭新形象。

　　深入实施城市绿化、亮化、美化、净化和道路畅通“五大工程”。实施城市绿化工程，实现立体绿化和绿化全覆盖，建成区绿化覆盖率达到39.2%，人均公园绿地面积达到9.3平方米，打造绿色生态锦州。实施城市亮化工程，实现主要街路立体亮化和中心城区亮化的全覆盖。实施城市美化工程，加大城市综合治理力度，美化城市环境。实施城市净化工程，逐步实现道路清扫、除雪和洗路等环卫设施机械化，主要街路24小时保洁。实施道路畅通工程，加强城市交通设施建设，实行交通严管，确保城市交通秩序井然、畅通安全。

　　加快推进城市基础设施和重点工程建设。突出抓好城市五大出口建设，全面启动生活垃圾和污泥焚烧发电厂、混合垃圾卫生填埋场等环保工程；切实加强城市道路交通网络建设，实现主次干道全部提档达标和小街小巷的全部硬覆盖；加快推进锦凌水库、城际铁路、广州街公铁立交桥、广州街小凌河大桥、中央南街立交桥、单洞扩孔等续建和新开工重点工程建设；加强给排水、集中供热和燃气管道建设，城市集中供热率、自来水和管道燃气普及率分别达到85%、97%和93%；加大棚户区改造力度，全面完成主城区棚户区改造任务；进一步增加廉租房、经济适用住房、公租房建设投入，满足贫困家庭和低收入群体住房需求，全年完成房地产开发面积1100万平方米，把锦州这座具有悠久历史和深厚文化底蕴的英雄城市打造成最宜人居、最宜创业、最具发展潜力和发展活力的城市，加快人流、物流、资金流、信息流的流动，不断提高对外知名度、吸引力，增强核心竞争力，推动锦州的快速发展。

　　大力发展以现代服务业为主体的城区经济。突出中央大街十里商街建设，高标准建设好地一大道、茂业百货、亿隆国际广场、家乐汇广场、温州名购、太阳广场城市综合体等重点项目，确保锦州国际酒店、国际会展中心7月投入使用，再引进一批国内外大型商贸企业，把中央大街打造成现代服务业集聚区。继续办好中国北方(锦州)农业新品种新技术展销会、中国古玩展等各类展会，繁荣商务会展业。加速渤海物流二期、恒大物流保税库区等工程建设，推动现代物流业蓬勃发展。加快推进七里河、大有、紫荆等温泉旅游开发项目，加速打造滨海大道旅游观光带和医巫闾山大旅游格局。全面提升现代服务业发展水平，努力把锦州打造成环渤海地区有凝聚力、影响力和辐射带动力的商贸购物中心、商务服务中心、休闲旅游中心和生态宜居中心。

　　(五)切实加强财税和金融工作

　　扩大经济总量，新增规模以上工业企业60户，培育新的经济增长点，形成新兴财源。培育纳税大户，重点培育税收超5000万元企业16户，其中亿元以上5户，壮大主体财源。提高经营城市水平，把城市作为最大国有资本经营，吸引外来资金和团队改造建设城市，增加后续财源。积极向上争取资金和项目，今年要确保向上争取资金12亿元，120个大项目列入国家和省计划，扩大补充财源。

　　加强金融生态环境建设，搭建融资平台，引进新的金融机构落户锦州。全市金融机构要抢抓国家稳健货币政策的机遇，利用我市存贷比空间，加大信贷投放力度，推动锦州快速发展。充分发挥商业银行及其分支机构和城投公司、小额贷款公司、村镇银行的融资作用。力促锦州银行、新华龙集团等企业成功上市。畅通融资渠道，化解金融风险，保障金融安全，完善借用管还的长效机制，确保锦州建设和发展的需要。

　　(六)大力发展以民生为重点的社会事业

　　一饭膏梁，维系万家；油盐柴米，关乎大局。我们要始终把百姓的衣食住行、安危冷暖挂在心上，把为百姓解难题、办实事、谋福祉作为全部工作的根本出发点和落脚点，深入实施民生工程，推动社会各项事业的全面建设、全面发展、全面进步。

　　优先发展教育事业。启动实施《锦州市中长期教育改革与发展规划纲要》，加强农村九年一贯制学校标准化建设，实施学前教育普及和基础教育强县(市)区建设工程，解决好进城务工人员子女入学和“留守儿童”学习问题，办好人民满意的教育。

　　突出发展卫生事业。继续推进医药卫生体制改革，实施国家基本药物制度。不断健全农村及社区卫生服务基础网络。加强疾病预防控制工作，提高应对突发公共卫生事件能力。进一步整合医疗卫生资源，切实加大“一个中心、多个专科”建设力度。加快医疗信息共享平台建设，实现医疗资源共享。深化人口和计划生育综合改革，加快完善农村部分计划生育家庭奖励扶助和特别扶助政策。

　　积极发展文化体育事业。不断深化文化体制改革，大力发展文化产业。认真抓好农村文化信息资源共享、乡镇综合文化站和农村电影“2131”工程。继续办好笔架山海洋音乐节等大型节庆文化活动，不断丰富城乡群众文化生活。切实加强城乡公共体育设施建设，推进全民健身和竞技体育的健康发展。

　　坚持就业是民生之本。深入开展创业型城市创建活动，认真做好普惠制就业培训和城镇实名制就业安置工作，进一步完善以零就业家庭为主体的困难群众就业援助机制，千方百计创造就业机会，确保零就业家庭继续保持动态为零，登记失业的困难家庭大学生全部实现当年就业，城镇登记失业率控制在3.5%以内，努力实现百姓充分就业、安居乐业。高度重视人才工作，加快建设面向辽西、功能齐全、设施完善的现代化人才市场。

　　不断完善社会保障体系。实现“五险合一”的全覆盖。全面启动居民医保大病门诊统筹。提高城乡低保补助水平。大力推进基本养老服务事业发展，养老机构新增床位500张，总量达到4000张。新增城市社区办公用房1万平方米，完成40个社区服务站建设，提高社区建设和管理水平。

　　全力维护社会和谐稳定。认真做好第九届城市居委会换届选举工作。进一步加强军政军民团结。切实做好民族宗教工作。全面启动“六五”普法，不断拓展法律服务领域。深化公安信息化建设，完善公共安全视频监控系统等基础设施，改善基层派出所办公条件，巩固提高社会治安防控体系建设水平，加快平安锦州建设进程。进一步健全领导包案和信访接待日制度，落实信访责任制，发挥信访大厅主体作用，有效化解各类社会矛盾。认真做好生产安全、食品安全、药品安全、交通安全、消防安全等10个方面的安全工作，切实维护人民的生命财产安全，确保全市政治安定、社会稳定。

　　(七)努力建设全市人民满意的政府

　　自觉接受人大法律监督、工作监督和政协的民主监督，坚持重大事项向人大报告和向政协通报制度，坚决执行人大及其常委会的各项决议、决定，积极支持人民政协履行职能，认真办理人大议案、代表建议和政协提案，切实做到民主决策、依法决策、科学决策。

　　深入贯彻《依法行政实施纲要》，健全依法行政各项制度，全面推行依法行政。完善政绩考核工作机制，深化行政审批制度改革，严格执行《政府工作规则》、《服务承诺制》、《首问责任制》、《限时办结制》等规章制度，加快推进公共服务平台建设，进一步优化经济发展环境。

　　始终坚持执政为民、务实清廉的从政准则，认真贯彻《党员领导干部廉洁从政若干准则》，严格执行8项禁止、52个不准，规范行政权力运行，从源头上防止腐败，塑造风清气正的政务环境，建设廉政勤政政府，用实实在在的工作成果取信于民、造福于民。

　　始终保持旺盛的斗志和昂扬向上的创业激情，大力弘扬“勇于牺牲、敢为人先”的锦州精神，重实际、重实干、重实效，一门心思抓发展，扑下身子抓落实，把全部时间和精力用在发展上，用在为全市人民谋福祉上，让政府工作经得起实践、群众和历史的检验，努力建设党和人民满意的政府。

　　各位代表，锦州取得的一系列巨大成就又让我们站在了一个新的起点，全市人民的热切期盼和“十二五”规划的宏伟蓝图激励着我们实现新的跨越。我坚信，有省委、省政府和市委的正确领导，有人大、政协和社会各界的监督与支持，有全市党政军民的共同奋斗，我们建设环渤海地区特大型港口城市、辽西沿海经济区中心城市、中国光伏产业之都和稳定进入全省第一方阵的目标一定会实现，锦州的明天一定会更加美好！
